# Effects of flight and food stress on energetics, reproduction, and lifespan in the butterfly *Melitaea cinxia*

**DOI:** 10.1007/s00442-019-04489-8

**Published:** 2019-08-22

**Authors:** Kristjan Niitepõld

**Affiliations:** 1 Faculty of Biological and Environmental Sciences, Metapopulation Research Centre, PO Box 65, Viikinkaari 1, 00014 University of Helsinki, Finland; 2 The Finnish Science Centre Heureka, Tiedepuisto 1, 01300 Vantaa, Finland

**Keywords:** Life-history, Migration, Oxidative stress, Senescence, Trade-off

## Abstract

Environmental change can have drastic effects on natural populations. To successfully predict such effects, we need to understand how species that follow different life-history strategies respond to stressful conditions. Here I focus on two stressors, increased flight and dietary restriction, and their effects on bioenergetics and life-history. Using the Glanville fritillary butterfly (*Melitaea cinxia*), I subjected mated females to three treatments: (1) control conditions, (2) repeated forced flight with unlimited food, and (3) repeated forced flight coupled with food restriction. Interestingly, flight increased fecundity: females in both flight treatments initiated oviposition earlier, laid more egg clutches, and had higher total fecundity than control females. However, food-restriction by 50% reduced clutch size and resulted in an approximately 25% decrease in total fecundity compared to flown females with unlimited food. There were no differences in egg wet mass, water content or hatching success. Flown females with unlimited food appeared to exhibit a trade-off between reproduction and lifespan: they had higher mass-independent resting metabolic rate and shorter lifespan than females in the other treatments. Mass-independent flight metabolic rate, reflecting flight capacity, did not differ among the treatments. There were no differences in the rate of metabolic senescence across the treatments. The current findings suggest a mechanistic link between flight and reproduction, potentially mediated by juvenile hormone signalling. It appears that this wing-monomorphic butterfly does not show an oogenesis-flight trade-off often found in wing-dimorphic insects. Nevertheless, nectar-feeding is needed for achieving maximum reproductive output, suggesting that diminishing nectar resources may negatively impact natural populations.

## Introduction

A key challenge in ecology is to predict effects of environmental change on natural populations. Most organisms are susceptible to short-term environmental variation and, in increasing amounts, large-scale environmental degradation due to processes such as anthropogenic climate change, habitat loss and fragmentation, and the spread of invasive species (Tilman et al. 2017). In order to understand changes at the population level, one way forward is to focus on performance and fitness at the individual level. Here, one condition that has received significant attention is stress caused by scarcity of food. Limited food availability is an ecologically relevant challenge that can affect population growth rate (Benton et al. 2005; Boggs and Inouye 2012), and have evolutionary consequences, such as selection for certain mitochondrial haplotypes apparently more resistant to starvation (Drovetski et al. 2012). Dietary restriction is also intensively studied in the context of ageing research, as it has been shown to extend lifespan in several species (Speakman and Mitchell 2011). How species respond to different stressors depends on their life-history strategy, but we still have a limited understanding of these responses, particularly when multiple stressors occur simultaneously, as is often the case in the wild.

Limited nutrient availability may lead to trade-offs between various processes, such as survival and reproduction (van Noordwijk and de Jong 1986). A useful tool to understand responses to food limitation is the resource allocation framework (Boggs 2009) that recognises that nutrients are allocated among many processes, including storage and investment in foraging. The latter is particularly interesting, as gaining more resources may require movement, which in itself is energetically demanding. Indeed, increased movement is a response that animals may use when resource availability is locally limited or the habitat is becoming increasingly fragmented (Clobert et al. 2009; Taylor and Merriam 1995).

Insect study systems have provided substantial insights into questions about trade-offs and resource allocation under stressful conditions (Clark et al. 2013; Lee et al. 2008; Roff and Fairbairn 2007; Zera and Harshman 2001), including transgenerational effects (Woestmann and Saastamoinen 2016). Due to the high energetic demands of flight (Suarez 2000), insects may succumb to oxidative damage (Pekny et al. 2018) or exhibit the so-called oogenesis-flight syndrome that arises when flight has a negative impact on reproduction (Johnson 1969). In the context of trade-offs, some of the most thorough investigations have concentrated on wing-dimorphic species, such as plant hoppers and crickets (Zera and Denno 1997). In contrast, we know much less about the relationship between flight and reproduction in wing-monomorphic taxa such as flies, mosquitoes, moths, and butterflies that typically have life styles that heavily depend on flight.

In insects, nutrient intake may significantly differ between the larval and adult stages, and specific nutrients may be used immediately or stored for later use (O’Brien et al. 2002). Some species are strict capital breeders, solely depending on larvally derived nutrients, and adults may even lack functional mouthparts (Tammaru and Haukioja 1996). Among Lepidoptera, many species use a mix of larval and adult resources for reproduction, and adult food restriction often has a negative effect on fecundity, such as in the case of the African fruit-feeding butterfly *Bicyclus anynana* (Bauerfeind and Fischer 2005; Geister et al. 2008), the Palearctic nectar-feeding butterfly *Maniola jurtina* (Lebeau et al. 2018), and the North American nectar-feeder *Speyeria mormonia* (Boggs and Ross 1993). Flight during early life has been shown to reduce egg size in the Palearctic speckled wood butterfly *Pararge aegeria* (Gibbs et al. 2010). Negative effects of flight may also carry over to the next generation. When reared on drought-stressed plants, offspring of flight-stressed speckled wood females had lower pupal masses compared to offspring of unstressed females (Gibbs et al. 2018). Flight may also negatively affect reproductive output via males, as in the case of the large white *Pieris brassicae,* whose males provide the female with an energetically expensive nuptial gift incorporated into the spermatophore that is transferred during copulation (Ducatez et al. 2012).

Differential allocation of nutrients is reflected in the energy consumption of animals. Dietary restriction has been shown to reduce resting metabolic rate (RMR), which represents the minimum maintenance cost of the physiological machinery (Djawdan et al. 1997; Nespolo et al. 2005; Niitepõld et al. 2014; Roark and Bjorndal 2009). Flight, on the other hand, increased RMR in the butterfly *Speyeria mormonia* (Niitepõld and Boggs 2015). Flight metabolic rate reflects flight capacity, and studies on the Glanville fritillary butterfly *Melitaea cinxia* have established a positive connection between flight metabolic rate and dispersal rate in females (Haag et al. 2005; Niitepõld et al. 2009). In contrast to RMR, flight metabolic rate appears to be conserved under dietary restriction in *Colias eurytheme* and *Speyeria mormonia* (Niitepõld et al. 2014). However, experimental flight treatments may lead to increased loss of function with age: the rate of metabolic senescence was accelerated in *Speyeria mormonia* females that were subjected to flight (Niitepõld and Boggs 2015).

Here, I addressed the effects of stress on energetics and life-history in the Glanville fritillary butterfly, focusing on increased flight and reduced food intake. The aim of the study was to find out how flight affects investment in reproduction, somatic maintenance, and longevity. One further aim was to see if allocation to these processes changes when food availability is limited. As the effects of stressful conditions on animal energetics are still poorly understood, I also wanted to know how resting and flight metabolic rates respond to different stressors, and whether there are effects on metabolic senescence. I performed an experiment where mated full-sib female Glanville fritillaries were subjected to three treatments: (1) control with unlimited food, (2) experimental flight coupled with unlimited food, and (3) experimental flight as in the previous treatment, but with a 50% reduction in the volume of food.

Even though many insects exhibit a trade-off between flight and reproduction, correlational evidence suggests that mobility and fecundity may be positively linked in the Glanville fritillary (Saastamoinen 2007). In *Speyeria mormonia*, experimental flight treatments led to earlier egg laying (Niitepõld and Boggs 2015). In this light, I predicted that flight would not negatively affect total fecundity, but it may affect the duration of the pre-oviposition period, at least in females that had unlimited access to food. I predicted that the Glanville fritillary places towards the capital breeder end of the larval vs. adult resources for breeding spectrum (O’Brien et al. 2004; Saastamoinen et al. 2013), and effects of adult food restriction would, therefore, not be as severe as in income-breeders such as *Speyeria mormonia*, which reduces its egg production roughly by 50% if subjected to 50% quantitative food reduction (Boggs and Ross 1993). I also predicted that flight would elevate RMR, whereas food restriction would reduce it, but that flight metabolic rate would not be affected by food restriction. Additionally, I predicted that flight would lead to faster senescence in flight metabolic rate, as in *Speyeria mormonia*, particularly in food-restricted females, but lifespan would not be affected by the treatments.

## Materials and methods

### Rearing of the butterflies

The experiment was performed on female Glanville fritillary butterflies. The species is univoltine in northern Europe. Females emerge with the full number of oocytes in their ovarioles and with at least some fully matured eggs (Boggs and Nieminen 2004). Adults feed on flower nectar and lay eggs in clutches on the host plants *Plantago lanceolata* and *Veronica spicata*. The parent generation was collected in the Åland Islands in SW Finland as larvae in the autumn of 2013 and reared until pupation at Lammi Biological Station. The parent generation mated and laid eggs in a large outdoor enclosure in the summer of 2014, and the larvae were reared in the laboratory where they were fed leaves of *Plantago lanceolata*. After winter diapause in controlled conditions at 4 °C, the larvae were reared in sibling groups until pupation under a 12:12 h light:dark cycle and a multistep 28:15 °C temperature cycle in the spring of 2015. After pupation, the individuals were placed in individual containers in which the adults emerged. Females were mated with unrelated males. The majority (72% of the females) mated on the first day after emergence, and the rest mated at ages 2 to 4 days. Females that had not mated by day 4 were excluded from the experiment. After mating, each female was housed in a cage that was placed over a potted host plant.

### Treatments

I used a split-brood design and assigned full-sib females from 10 families to three treatments: control, flight with ad lib feeding (flight), and flight with ½ ad lib feeding (flight + DR). When more than three females per family were available, I assigned additional triplets to the treatments. In total 56 females were used in the experiment (3–9 females from each family). There were no statistically significant differences in body mass among females in the groups in the beginning of the experiment (*F*_2,53_ = 1.05, *p* = 0.36). The controls were fed twice a day with 20% honey–water solution provided as a droplet on a petri dish while the individual was held from its wings and its proboscis was extended into the droplet. Once the butterfly started feeding, its wings were released and it was allowed to feed ad libitum. To determine its food intake, the individual was weighed before and after feeding with a Mettler Toledo XS 105 scale (Mettler Toledo, Greifensee, Switzerland). Females in the flight treatment were forced to fly in a cylinder cage (40 by 50 cm) as continuously as possible in four bouts of 4 min, by disturbing them with a paintbrush. The treatment was performed on seven consecutive days, beginning from the day after mating. In between the 4-min flight treatments, there was a 5-min period of rest. The flight females were fed in the same way as controls. Females in the flight + DR treatment were flown as above, but they received only 50% of the amount of honey water consumed by their sister in the flight treatment at the same age. The exact amount of honey water was provided with a pipette on a petri dish while the butterfly was held from its wings as in the other treatments.

The cages of all females were checked for eggs daily. The eggs were collected, counted, and their wet mass was weighed using a Mettler Toledo XS 105 scale. The first clutch of each female was transferred on a Petri dish with lightly moist filter paper. The eggs were allowed to hatch and the hatching success was calculated in per cent. To determine the dry mass of eggs, I dried the subsequent egg clutches at 50 °C for 3 days, and weighed the eggs using a Mettler AE163 scale (Mettler Toledo, Greifensee, Switzerland, reproducibility 0.02 mg).

I measured the resting and flight metabolic rates of each female at ages 3, 9, and 15 days using flow-through respirometry (see Niitepõld et al. (2014) for details on the methods). Four females had not been assigned to their treatments at the time of the measurement on day 3. These data points were excluded from the statistical analyses. The females were measured in the morning and they had received their latest meal in the afternoon of the previous day, on average 17 h 16 min prior to the measurement (SD 1 h 41 min, range 13 h 34 min–21 h 24 min). In short, I placed the butterfly in a 1–l measurement chamber that I covered with a black cloth and allowed the individual to rest for about 25 min before the start of the measurement. I used the mean of 1.5 min of stable CO_2_ production for the calculation of RMR. Butterflies tend to sit still when kept in the dark, and if an individual moved, this was easily seen from the real-time CO_2_ recording, and I allowed the individual to settle to rest. The temperature inside the chamber was continually measured with a thermistor probe. The mean across RMR measurements was 31.9 °C ± SD 0.7 (min = 30.5, max = 33.4). The temperature corresponds to optimal body temperature for flight in *M. cinxia* (Niitepõld 2010). To measure flight metabolic rate, I removed the black cloth and exposed the butterfly to light. After 30 s I began to shake the chamber in order to encourage the butterfly to fly as continuously as possible during the 7 min measurement. I extracted two parameters from the recording: peak flight metabolic rate and total volume of CO_2_ produced during flight. The first parameter reflects maximum flight capacity, and the latter overall flight performance and endurance. The mean temperature across measurements of flight metabolic rate was 31.9 °C ± 0.6 (min = 30.1, max = 33.0). After the measurement, I weighed the butterfly and gave it the first honey-water meal of the day (as above).

### Statistical analyses

Body mass was analysed using a repeated measures model (repeated statement with female as subject) with autoregressive (1) covariance structure in Proc Mixed in SAS 9.4. The dependent variable was body mass in the morning at ages 1, 2, 5, 8, 11, 14, 17, and 20. Family and treatment were added as independent class variables, and age as a continuous variable. The model also contained age squared to detect nonlinear age effects, as well as the interaction between age and treatment. Food intake in the morning and afternoon were modelled using repeated measured models with family and treatment as class variables and age as a continuous variable. The models also contained age squared and the interaction between age and treatment. The metabolic rates were modelled with repeated measures models with family and treatment as class variables and age (3, 9, and 15) as continuous variables. Temperature inside the chamber was added as a covariate, but removed if nonsignificant (*p *> 0.05). The probability of laying any eggs vs no eggs was modelled using logistic regression. Total number of eggs (excluding zeroes) was modelled using a generalised linear model (Proc Glimmix) with Poisson error structure. Body mass and lifespan were included as covariates in the model. Family and treatment were included as factors. Age at the onset of oviposition, number of clutches (excluding females that laid no eggs), and lifespan were modelled with gamma error structure. Clutch size in all clutches laid was analysed with a repeated measures model that included family and treatment as class variables and age as a covariate. Following inspection of the results, a second model was constructed that contained only the first clutches of each female that laid eggs. Egg hatching success (of the first egg clutch) was modelled using ANCOVA. Egg wet mass and egg water percentage were modelled with linear mixed models with female as a random factor, as all clutches except for the first clutch of each female were included in this analysis.

## Results

### Body mass

**Fig. 1:**
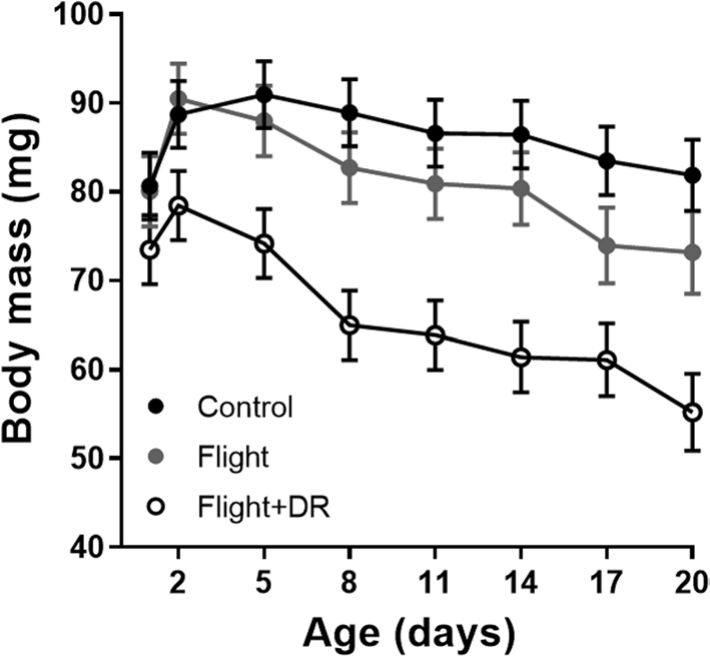
Body mass plotted against age. Food-stressed females (open circles) had the lowest body masses throughout the experiment. Black circles represent control females and grey circles females from the flight treatment with unlimited food. Symbols display means with standard errors

Body mass was measured repeatedly during the entire adult lifespan. There were significant differences in body mass among the treatments (*F*_2,44_ = 6.99, *p* = 0.002) (Table 1). Flight + DR females were lightest, while the body masses of control and flight treatment females were not statistically distinguishable from each other (Fig. 1). Body mass was strongly affected by age: body mass first increased, then decreased (main effect: *F*_1,334_ = 16.16, *p* < 0.0001; age^2^: *F*_1,334_ = 3.77, *p* = 0.05). Females in the flight + DR treatment, however, diverged from this pattern, as indicated by the significant treatment by age interaction (*F*_2,334_ = 4.60, *p* = 0.011). These females only showed a minor increase in body mass in the first days, and their body masses decreased throughout their lives. The effect of family was not significant (*F*_9,44_ = 1.31, *p* = 0.26) (Table 2).

**Table 1:** Summary table of repeatedly measured life-history traits

Trait	Age	Control (*n* = 19)	Flight (*n* = 19)	Flight + DR (*n* = 18)
		Mean ± SD	Mean ± SD	Mean ± SD
Body mass (mg)	1	82.0 ± 14.7	81.3 ± 14.2	75. 4 ± 16.5
	3	91.8 ± 16.3	96.6 ± 14.1	82.8 ± 16.5
	9	89.9 ± 19.3	84.2 ± 17.4	69.8 ± 16.7
	15	87.8 ± 18.93	84.8 ± 15.9	64.8 ± 10.7
Food intake in morning (mg)	3	8.29 ± 4.7	7.2 ± 4.9	3.7 ± 1.3
	9	5.1 ± 2.9	6.7 ± 3.9	3.5 ± 1.8
	15	3.10 ± 2.3	3.2 ± 2.6	1.87 ± 1.1
Food intake in afternoon (mg)	1	14.6 ± 7.0	16.0 ± 6.0	8.6 ± 4.66
	3	3.9 ± 2.6	2.9 ± 2.4	2.7 ± 3.0
	9	4.4 ± 3.3	3.4 ± 4.4	1.2 ± 1.3
	15	1.99 ± 1.47	3.2 ± 3.7	1.33 ± 1.0
RMR, ml CO_2_ h^−1^	3	0.138 ± 0.04	0.154 ± 0.03	0.125 ± 0.04
	9	0.107 ± 0.02	0.109 ± 0.02	0.084 ± 0.02
	15	0.100 ± 0.03	0.112 ± 0.02	0.074 ± 0.02
Mass-specific RMR, ml CO_2_ h^−1^g^−1^	3	1.50 ± 0.28	1.59 ± 0.23	1.50 ± 0.31
	9	1.22 ± 0.3	1.33 ± 0.3	1.23 ± 0.29
	15	1.17 ± 0.31	1.33 ± 0.22	1.13 ± 0.22
Peak MR, ml CO_2_ h^−1^	3	2.24 ± 0.51	2.33 ± 0.58	2.121 ± 0.50
	9	1.61 ± 0.50	1.42 ± 0.70	1.30 ± 0.43
	15	1.26 ± 0.31	1.21 ± 0.558	1.21 ± 0.45
Mass-specific peak MR, ml CO_2_ h^−1^g^−1^	3	24.7 ± 4.9	24.2 ± 5.2	25.5 ± 4.2
	9	18.2 ± 5.1	17.0 ± 7.2	19.4 ± 6.2
	15	14.9 ± 4.0	14.2 ± 5.77	18.8 ± 6.2

Mass-specific metabolic rates are presented for illustrative purposes. In the analyses, body mass was accounted for statistically

**Table 2:** Summary table of life-history data

Trait	Control (*n* = 19)	Flight (*n* = 19)	Flight + DR (*n* = 18)
	Mean ± SD	Mean ± SD	Mean ± SD
Age at onset of oviposition (days)	12.4 ± 5.6^a^	7.3 ± 2.5^b^	6.7 ± 3.3^b^
Number of clutches	1.6 ± 1.1^a^	2.25 ± 1.5^b^	2.7 ± 1.5^b^
Total number of eggs	259 ± 224^a^	392 ± 237^b^	291 ± 135^c^
Clutch size	176 ± 81^a^	174 ± 72^a^	112 ± 63^b^
Egg wet mass (mg)	0.08 ± 0.01^a^	0.08 ± 0.01^a^	0.09 ± 0.01^a^
Egg water content (%)	27.7 ± 0.7^a^	26.8 ± 1.8^a^	26.0 ± 1.8^a^
Hatching success (%)	47 ± 33^a^	56 ± 21^a^	65 ± 26^a^
Lifespan (days)	22.4 ± 5.7^a^	18.4 ± 4.4^b^	21.0 ± 6.2^a^

Letters represent statistically significant differences among treatments

### Food intake

The effect of treatment on food intake in the morning was significant, as expected, because females in flight + DR treatment only received 50% of the amount that their sister in the flight treatment consumed (Table 1). Statistics are presented in Table 3a. There was no difference in the intake of females in the flight treatment and control females (Tukey *p *= 0.27). Females ingested the largest volumes during the first day of feeding and intake decreased with age. This pattern resulted in a strongly concave age effect in all treatments.

**Table 3:** Statistical results for honey-water intake

Effect	(a) Morning intake			(b) Afternoon intake		
	*DF*	*F*	*p*	*DF*	*F*	*p*
Family	9, 44	1.29	0.27	9, 44	2.66	0.015
Treatment	2, 44	5.76	0.0060	2, 44	9.30	0.0004
Age	1, 281	59.96	< 0.0001	1, 328	108.57	< 0.0001
Age × treatment	2, 281	0.61	0.55	2, 328	1.55	0.21
Age^2^	1, 281	28.90	< 0.0001	1, 328	55.66	< 0.0001

Both models were repeated measures mixed models with autoregressive (1) covariance structure

Food intake in the afternoon was qualitatively similar to the morning intake with a strong effect of treatment, and a nonlinear age effect (see Table 3b for statistics). There was no difference between the intake of control and flight treatment females (Tukey *p *= 0.99).

### Metabolic rates

**Fig. 2:**
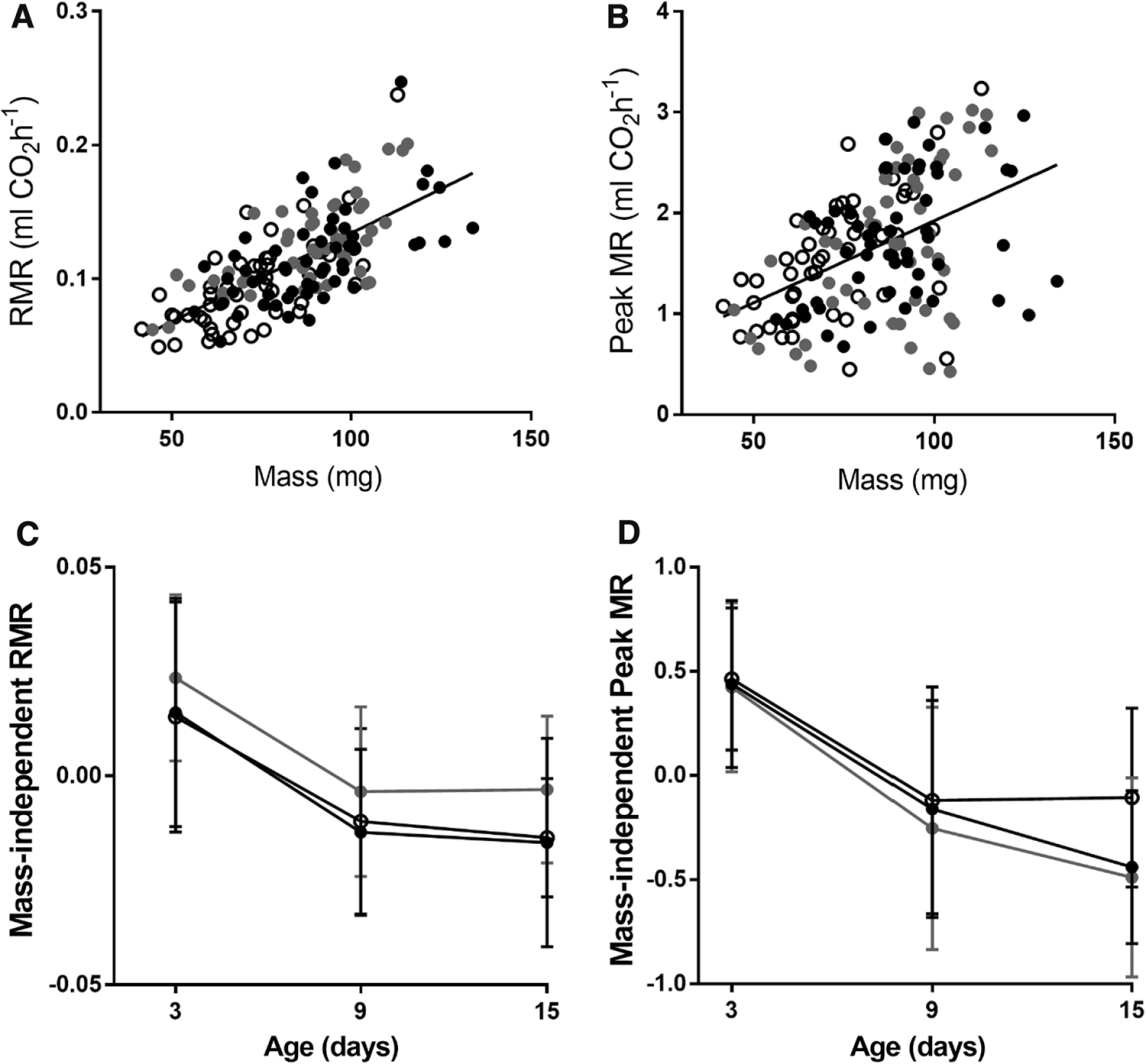
**a** Resting metabolic rate against body mass. Each female was measured three times. **b** Peak flight metabolic rate against body mass. **c** Mass-independent resting metabolic rate (residuals from a linear model accounting for body mass) plotted against age. The mean is shown for each group with standard deviation as the error bar. Black circles represent control females, grey circles the flight treatment, and open circles the flight + DR treatment. Flight treatment females had the highest resting metabolic rates. **d** Mass-independent peak flight metabolic rate plotted against age. There were no significant differences among the treatments

Body mass had a positive effect on RMR (Fig. 2a). When body mass was accounted for, RMR differed among the three treatments: females in the flight treatment had the highest mass-independent RMR, while control females and flight + DR females could not be distinguished from each other (Fig. 2c). Statistics are presented in Table 4a. RMR decreased strongly with age, and the decrease was strongest from the first time point to the second. Measurement temperature had a positive effect on RMR.

**Table 4:** Statistical results for metabolic rates

Effect	(a) RMR			(b) Peak MR			(c) Total CO_2_		
	*DF*	*F*	*p*	*DF*	*F*	*p*	*DF*	*F*	*p*
Family	9, 44	4.28	0.0005	9, 44	1.6	0.14	9, 44	1.52	0.17
Treatment	2, 44	21.97	< 0.0001	2, 44	1.03	0.37	2, 44	1.99	0.15
Body mass	1, 88	106.93	< 0.0001	1, 89	38.51	< 0.0001	1, 89	23.0	< 0.0001
Age	1, 88	51.31	< 0.0001	1, 89	99.56	< 0.0001	1, 89	113.88	< 0.0001
Temperature	1, 88	9.79	0.0024	–	–	–	–	–	–
Age*Treatment	2, 88	0.02	0.98	2, 89	0.94	0.40	2, 89	0.99	0.38
Age^2^	1, 88	16.63	< 0.0001	1, 93	14.37	0.0003	1, 89	22.16	< 0.0001

Temperature was not included in the final models for Peak MR and Total CO_2_ production as it was not significant. All models were repeated measures mixed models with autoregressive (1) covariance structure

Body mass had a positive effect on peak flight metabolic rate (Fig. 2b, Table 4b). Treatment had no effect on peak flight metabolic rate (Fig. 2d). Peak flight metabolic rate showed a strong decrease with age. There were no differences in the rate of metabolic senescence as indicated by the non-significant age by treatment interaction. After visual inspection of the data, I added an interaction between the quadratic age term and treatment in the model, but as it was non-significant, I did not include it in the final model.

Females in the three treatments did not differ in the total output volume of CO_2_ (Table 4c). Body mass had a positive effect, and there was a strong decrease with age that followed a nonlinear pattern. The age by treatment interaction was not significant.

### Egg production

**Fig. 3:**
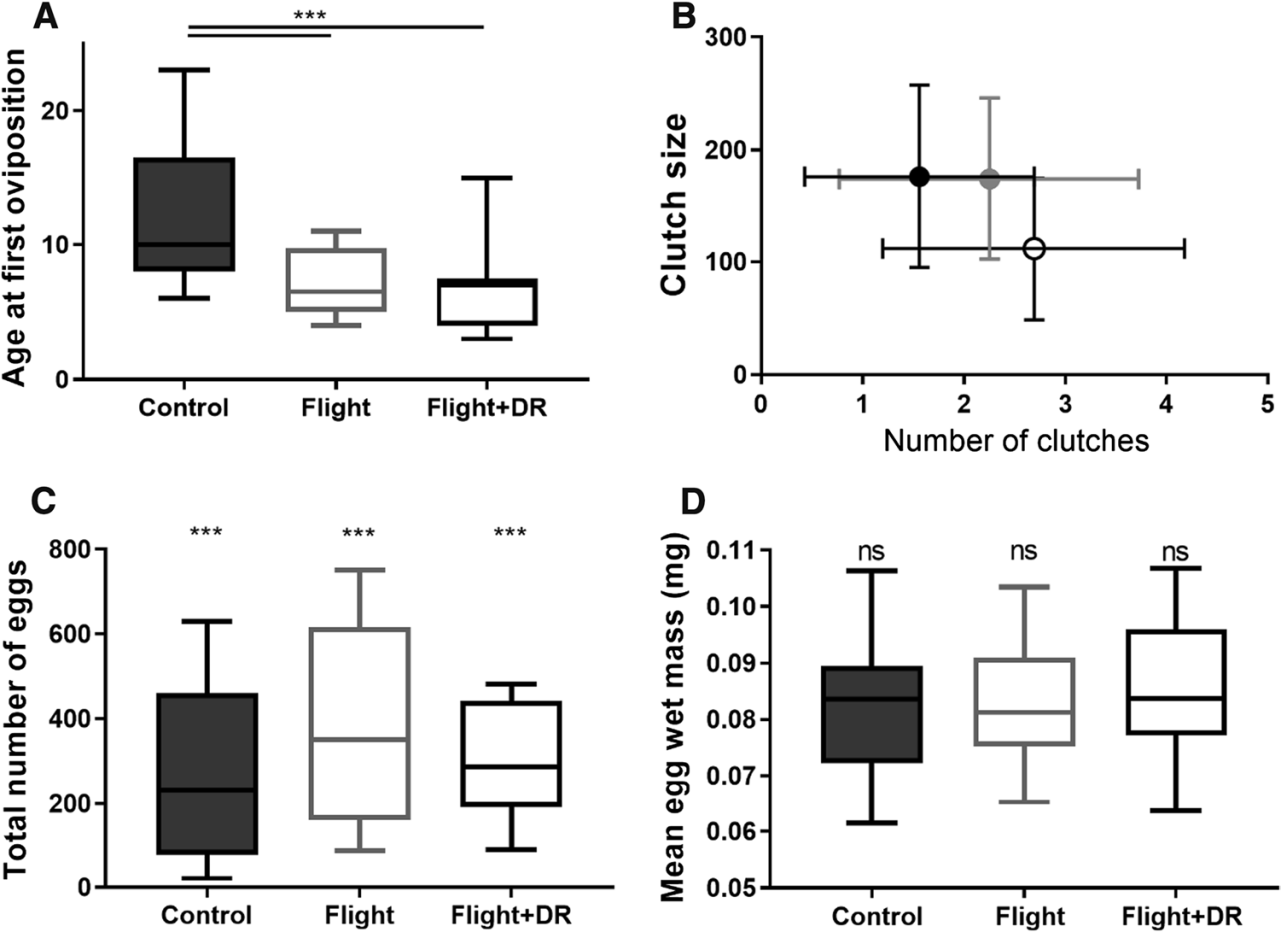
**a** Age at first oviposition in the control, flight treatment and flight + DR treatment. The horizontal lines in the boxplots depict the median, and outlines of the boxes 25th and 75th percentiles. Whiskers depict minimum and maximum values. Only females that laid any eggs are included. The proportion of females that laid no eggs was 10/19 in the control, 7/19 in the flight treatment, and 5/18 in the flight + DR treatment. **b** Mean egg clutch size against mean number of egg clutches. The black filled circle represents the control, the grey circle the flight treatment and the open circle the flight + DR treatment. The whiskers indicate standard deviations. **c** Total number of eggs laid during the lifetime. Only females who laid eggs were included in the analysis. **d** Mean wet mass of eggs. There were no significant differences among the three treatments

The age at which females laid their first eggs was affected by the treatments: females in both stress treatments started laying eggs earlier than control females (*F*_2,23_ = 3.97, *p* = 0.006). Flight females laid their first eggs on average 5 days earlier, and flight + DR females closer to 6 days earlier than control females (Fig. 3a) (Table 2). The age at first oviposition did not statistically differ between the two stress treatments (Tukey *p* = 0.96). The effect of family was statistically significant (*F*_7,23_ = 21.97, *p* = 0.002). The mass of the female on the first full day after emergence was used as a covariate, but the effect was not significant (*F*_1,23_ = 1.69, *p* = 0.21).

The probability of laying eggs did not differ among the treatments, though there was a trend towards stressed females having higher probability of laying eggs than control females (*F*_2,43_ = 2.39, *p* = 0.10). The effect of family was not significant (*F*_9,43_ = 1.11, *p* = 0.38), and body mass had no significant effect on egg-laying probability (*F*_1,43_ = 0.38, *p* = 0.54).

The number of egg clutches was higher in the flight + DR (median 3 clutches) and flight treatment (median 2) compared to the control (median 1 clutch) (*F*_2,23_ = 8.51, *p* = 0.002) (Fig. 3b). The number of clutches did not differ between the two stress treatments (Tukey *p* = 0.22). The effect of family was significant (*F*_7,23_ = 4.13, *p* = 0.005). Female mass had a positive effect on clutch number (*F*_1,23_ = 9.21, *p* = 0.006).

The number of eggs in the first clutch laid by each female did not differ between the treatments (*F*_2,23_ = 2.05, *p* = 0.15), whereas the effect of family was significant (*F*_7,23_ = 2.61, *p* = 0.039). A repeated measures model, however, showed that across all clutches, treatment had a significant effect (*F*_2,23_ = 7.43, *p* = 0.003) (Fig. 3b). Females in the flight + DR treatment laid smaller clutches than females in the other treatments (Table 2). Clutch size decreased with age (*F*_1,44_ = 14.73, *p* = 0.0004), and the effect of family was significant (*F*_7,23_ = 2.65, *p* = 0.036).

The total number of eggs was affected by the treatments: control females laid fewer eggs than stressed females (*F*_2,20_ = 8.77, *p* = 0.0018) (Fig. 3c). Compared to control females, flight + DR females laid 12% more eggs, and flight females 51% more eggs (Table 2). A Tukey’s test indicated that all treatments differed significantly from each other (*p* < 0.0001). There was a significant effect of family on the total number of eggs (*F*_7,20_ = 129.6, *p* < 0.0001), and heavier females laid more eggs than lighter females (*F*_1,20_ = 272.8, *p* < 0.0001). Lifespan was positively correlated with the total number of eggs (*F*_1,20_ = 49.97, *p* < 0.0001). A significant treatment by lifespan interaction (*F*_1,20_ = 34.37, *p* < 0.0001) indicated that the slope between lifespan and egg production was steepest in the flight treatment.

### Egg quality

The wet mass of eggs did not differ among the three treatments (*F*_2,23_ = 2.49, *p* = 0.11) (Fig. 3d). Female mass in the morning of the day of oviposition had a positive effect on wet egg mass (*F*_1,35_ = 21.00, *p* < 0.0001). There was a significant effect of family (*F*_7,23_ = 12.61, *p* < 0.0001).

The percentage of water in eggs was not affected by the treatments (*F*_2,16_ = 0.31, *p* = 0.74). Female mass had no significant effect on egg water percentage (*F*_5,16_ = 0.40, *p* = 0.53). The effect of family was significant (*F*_5,16_ = 3.16, *p* = 0.036). The number of eggs in the cluster had a highly significant positive effect on water percentage (*F*_1,16_ = 31.43, *p* < 0.0001).

There was a large amount of variations in hatching success of eggs (from 7 to 97%), but hatching success did not differ between the treatments (*F*_2,15_ = 0.63, *p* = 0.55). The effect of family on hatching success was not significant (*F*_7,15_ = 0.19, *p* = 0.98). There was no effect of the mass of the female (*F*_1,15_ = 0.08, *p* = 0.78) or the average egg wet mass (*F*_1,15_ = 0.44, *p* = 0.52) on hatching success.

### Lifespan

**Fig. 4:**
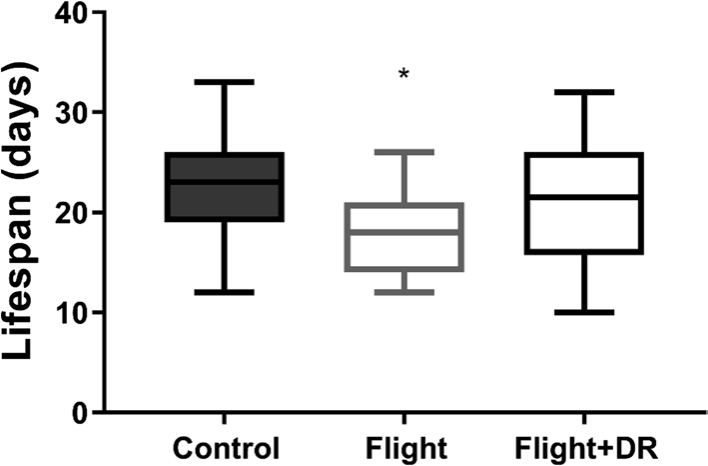
Lifespan in the three treatments. Females in the flight treatment with unlimited food had significantly shorter lifespans than control females or females that experienced forced flight and dietary restriction

Females in the flight treatment had shorter lifespans than control females or females in the flight + DR treatment (Table 1) (*F*_2,43_ = 3.36, *P* = 0.044) (Fig. 4). The effect of family was not significant (*F*_9,43_ = 6.72, *P* = 0.69), but there was a nonsignificant trend of larger females living longer (*F*_1,43_ = 2.64, *P* = 0.11).

## Discussion

Understanding effects of environmental change on natural populations is a fundamental challenge in ecology in the twenty-first century. Here, I examined the effects of repeated flight and chronic food stress on reproduction, lifespan and other critical life-history traits in the ecological model species *Melitaea cinxia*. I found strong effects of dietary restriction on body mass, RMR, and total fecundity. Flight, on the other hand, increased investment in reproduction, which together with previous findings suggests that wing-monomorphic insects, such as butterflies may not trade off fecundity against flight (Niitepõld and Boggs 2015; Saastamoinen 2007; Sappington and Showers 1992). However, flight shortened lifespan in females with unlimited food. Females thus appeared to follow different strategies in the three treatments: control females had low RMR, low reproductive output and long lifespans, whereas flight females had high RMR, high reproductive output and short lifespans. Flight + DR females had elevated reproductive output, but no increase in RMR or decrease in lifespan, consistent with positive effects of dietary restriction as reported in several other species. Neither dietary restriction nor experimental flight treatments affected flight metabolic rate or the rate of senescence in flight metabolism. These results may reflect adaptation to a lifestyle where flight has obligatory functions during daily activity within a habitat patch and during dispersal among scattered habitat patches, both being critical for metapopulation dynamics (Hanski et al. 2017).

### Effects on reproduction and lifespan

The most striking finding regarding reproduction was that flight-stressed females increased investment in reproduction. Compared to control females, females in the flight and flight + DR treatments started laying eggs at a younger age, laid more clutches, and had higher total fecundity. I had predicted that flight would shorten the pre-oviposition period, but the increase in the total number of eggs was unpredicted. This finding may appear surprising in the light of life-history theory, as we expect there to be a trade-off between energetically expensive processes such as flight and reproduction (van Noordwijk and de Jong 1986). Despite the elegant investigations on the oogenesis-flight syndrome in crickets and other wing-dimorphic insects, the trade-off is not necessarily a general phenomenon. Examples of positive relationships between flight and reproduction are well documented particularly among wing-monomorphic insects that use flight throughout their lives (Johnson 1969; Sappington and Showers 1992; Zera and Brisson 2012). Flight has been shown to advance the onset of reproduction for example in the diamondback moth *Plutella xylostella* (Johnson 1969), the rice leaf roller moth *Cnaphalocrocis medinalis* (Zhang et al. 2015), and in the Mormon fritillary butterfly (Niitepõld and Boggs 2015). Flight increased the rate of oocyte maturation also in the desert locust *Schistocerca gregaria* and the migratory locust *Locusta migratoria* (Highnam and Haskell 1964).

In the Glanville fritillary, correlational studies under semi-natural conditions have established that mobility is positively correlated with lifetime reproductive output in females from newly established populations and that mobile females initiate oviposition earlier than less mobile females (Saastamoinen 2007). Compared to females from old populations, females from newly established populations show higher mobility, higher juvenile hormone titers and an earlier onset of reproduction than (Wheat et al. 2011). The current study suggests that flight itself may play a causal role in increased investment in reproduction, most likely through juvenile hormone signalling. Indeed, in the grasshopper *Melanoplus sanguinipes* flight until exhaustion elevated juvenile hormone levels and shortened the pre-oviposition period, and a similar reduction in the pre-oviposition period was seen after topical application of juvenile hormone (Min et al. 2004).

How exactly flight affects reproduction may depend on the timing and duration of flight. Species differ in how mature their eggs are at the time of emergence (Wheeler 1996), and Glanville fritillary females emerge with the full number of oocytes in their ovarioles and a portion of the eggs are already mature at emergence, as in other checkerspot butterflies (Boggs and Nieminen 2004). In contrast, work on the speckled wood butterfly *Pararge aegeria* by Gibbs et al. (2010, 2018) demonstrated that an experimental flight treatment consisting of only 5 min of flight at the age of 1 day after emergence reduced reproductive success (egg size and early-life reproductive output), possibly because flight interfered with reproductive maturation. This interpretation is supported by work on the beet armyworm *Spodoptera exigua* that showed that flight had no significant effect on reproduction, except when moths were flown at the age of 1 day (Jiang et al. 2010). However, it is not well established which particular stage of egg production poses the greatest energetic cost. We know that RMR is at its highest in the first days after emergence and this could reflect the cost of egg construction. Glanville fritillaries show reduced flight performance and are reluctant to fly and on the first day after emergence (Niitepõld and Hanski 2013), which may reflect a behavioural mechanism for avoiding interfering with egg maturation.

Food restriction reduced egg production in flown females, but the percentual reduction was smaller than the reduction in food intake. This finding likely reflects the reproductive strategy of the Glanville fritillary where females use a mix of larval and adult-derived resources for reproduction and females already have some mature eggs in their ovarioles at emergence. Even though food-restricted females were unable to gain mass during early life, their egg production capacity was not strongly affected. In contrast, adult food restriction may have paramount effects on species that rely heavily on adult-derived nutrients for egg production, such as the above-mentioned *Pararge aegeria,* whose unfed females only laid less than a third of the eggs compared to fed females (Karlsson and Wickman 1990). In *Speyeria mormonia* as much as 80% of the carbon in eggs is of adult origin (O’Brien et al. 2004), and the proportion of adult-derived carbon decreases due to adult food restriction, and increases if females are subjected to experimental flight when fed ad lib (Boggs and Niitepõld 2014). Consequently, adult dietary restriction dramatically reduces fecundity (Niitepõld et al. 2014).

Interestingly flight shortened lifespan in flown females, but noticeably this effect was only seen in females with unlimited food. Food-stressed individuals had lifespans that were indistinguishable from those of control females. Here, the result resembles those from studies finding improved health and extended lifespans in organisms subjected to dietary restriction (Piper et al. 2011; Speakman and Mitchell 2011). The result suggests that adult lifespan is a plastic trait that may depend on the interplay among several processes, including investment in reproduction. Apparently flown females with unlimited food adopted a fast life-history strategy that allowed them to invest heavily in reproduction, seemingly at the expense of longevity. Such a strategy allows exploiting abundant resources by producing the offspring early which is bound to be advantageous in an organism that under all circumstances is short-lived. Flown food-restricted females, too, showed increased investment in reproduction, but they appeared to be somewhat energetically limited, which resulted in more frequent laying of small egg clutches. The lower clutch sizes were partly compensated by the increased lifespan in comparison to the flown females with unlimited food.

How flight affects longevity may also depend on previously experienced stress, such as in the case of the butterfly *Bicyclus anynana* where a one-time flight treatment resulted in shorter lifespan in females that were reared in a benign environment as larvae (Saastamoinen et al. 2010). In contrast, individuals that had experienced food-stress as larvae tolerated flight better. In the butterfly *Pararge aegeria*, flight has been shown to shorten lifespan in individuals originating from woodlands (Gibbs et al. 2018), but individuals from agricultural landscapes seem not to be negatively affected by flight (Gibbs and Van Dyck 2010).

The current results contradict results from an earlier experiment on the Glanville fritillary where a 15-min flight treatment had no effect on lifespan (Woestmann et al. 2017). Also, in the Mormon fritillary, daily flight treatments of 3 × 4 min had no effect on lifespan (Niitepõld and Boggs 2015). The exact physiological processes that underlie changes in lifespan are poorly known in butterflies, but studying the hormonal responses to different flight treatments would be a potential way forward. In insects, adipokinetic hormones that regulate fuel transport and metabolism serve an important part in the stress response (Gäde et al. 1997; Zemanová et al. 2016), and would be worth exploring in this context.

### Effects on metabolic rates

Patterns seen in RMR are among the key findings in this study. Noticeably, the experimental flight treatment elevated RMR when body mass was taken into account. This result resembles previous findings in the Mormon fritillary butterfly (Niitepõld and Boggs 2015) and is likely to reflect increased physiological activity due to flight and reallocation of resources. The high mass-independent RMR was only found in females with unlimited food; females in the flight + DR treatment had RMR that were at the same level as in control females with no flight. This pattern would suggest that females in the flight treatment with unlimited food switched to a metabolically faster lifestyle with high fecundity and shorter lifespan.

A lower rate of CO_2_ production in flight + DR females in comparison to the flight treatment females could also reflect switching from carbohydrate to lipid metabolism, which leads to a lower respiratory quotient (rq; the ratio between CO_2_ released in relation to O_2_ consumed) (Sinclair et al. 2011), but we have a limited understanding of the extent non-migratory butterflies utilise lipids for energy production. However, flight in the Glanville fritillary is solely fuelled by carbohydrates (rq = 1) (Haag et al. 2005), similar to the case of some other insects, such as bees (Suarez et al. 2005). Starvation was shown to reduce circulating glucose and trehalose levels in the Glanville fritillary, but no significant change in haemolymph triglyceride levels was found (Fountain et al. 2016). These findings suggest that while lipids have an important role in egg production, lipid oxidation may not be used for energy production in this non-migratory species, and the lower CO_2_ production would thus indicate a genuinely lower RMR.

Both mass-independent peak metabolic rate and the total volume of CO_2_ emitted during the measurement declined with age, reflecting metabolic senescence. This result is in agreement with the general notion of loss of performance with advancing age (Nussey et al. 2008; Partridge and Gems 2006), and findings such as reduced flight endurance in ageing *Pieris napi* butterflies (Ahman and Karlsson 2009) and reduced flight performance with age in *Drosophila melanogaster* (Miller et al. 2008). An age-related decline in flight metabolic rate was also seen in the butterflies *Colias eurytheme* and *Speyeria mormonia* under laboratory conditions similar to the current experiment (Niitepõld and Boggs 2015; Niitepõld et al. 2014).

I had predicted that forced flight would affect flight metabolic rate through accelerated metabolic senescence, but the relationship between flight metabolic rate and age did not differ among the treatments. The lack of an effect of forced flight on the rate of metabolic senescence differs from previous work on the butterfly *Speyeria mormonia* (Niitepõld and Boggs 2015) and *Drosophila melanogaster* (Lane et al. 2014). It is unknown whether the Glanville fritillary would be more tolerant to flight, or if the flight treatment represented a milder form of stress than in the other studies, but the result is consistent with the notion that butterflies appear to conserve their maximum flight capacity under stressful conditions (Niitepõld et al. 2014). Flight is crucial for all activities in the life of a butterfly, including dispersal to potentially more favourable habitats.

How food stress at the adult stage affects flight capacity is likely to depend on the severity of food restriction and the type of flight that is being measured as well as differences in life-history strategies. For example, 2-day-old *Lycaena tityrus* butterflies that had received no food after emergence showed reduced flight endurance in an intense shaking test (Reim et al. 2018). Similarly, sugar or blood fed mosquitoes *Anopheles gambiae* and *A. atroparvus* flew longer distances in a flight mill than did unfed controls (Kaufmann and Briegel 2004). Meadow brown butterflies *Maniola jurtina* kept under conditions that mimicked natural variation in nectar availability showed the highest flight metabolic rates when having access to the highest quantity of high-quality nectar (Lebeau et al. 2016). Glanville fritillaries typically fly in short bouts and effects of dietary restriction would almost certainly be different in butterflies in the act of performing long-distance migration where fat reserves play a large role (Dudley and Srygley 2008).

## Conclusions

The current study shows that adult Glanville fritillaries may be surprisingly resilient to stressors such as increased flight and reduced food intake. Indeed, flight increased reproductive output, and food stress appeared to guard against negative effects on lifespan. The study suggests that there is no clear trade-off between flight and reproduction in this wing-monomorphic butterfly under the conditions tested here. Curiously, it seems like a sedentary lifestyle and unlimited access to food were the most stressful treatments in terms of reproductive performance. The low reproductive output of ‘couch potato’ females in comparison to the more fecund flight-treated females resembles hormetic effects seen in vertebrates where a mild stressor may increase health and fecundity (Gems and Partridge 2008; Zhang et al. 2018), but to which extent mechanisms behind both observations are the same, calls for further study. Previous field studies on the Åland Islands metapopulation of the Glanville fritillary have identified environmental conditions during the larval stage as the most important drivers of population growth rate, rather than conditions during the relatively short adult period (Kahilainen et al. 2018; Tack et al. 2015). The current study confirms that larval resources have a significant role in reproduction, but the results also show that maximum reproductive potential cannot be reached without sufficient adult feeding in this species. Vulnerability to environmental variation at specific life-history stages highlights the importance of understanding species’ reproductive strategies when predicting effects of environmental change or making habitat management decisions.

## Data Availability

The datasets generated during and/or analysed during the current study are available in the Dryad data repository, 10.5061/dryad.m54ff05.
